# Osteoarthritis medical labelling and health-related quality of life in the general population

**DOI:** 10.1186/s12955-014-0146-8

**Published:** 2014-11-30

**Authors:** Sara Lourenço, Raquel Lucas, Fábio Araújo, Mónica Bogas, Rui André Santos, Henrique Barros

**Affiliations:** Institute of Public Health of the University of Porto, Rua das Taipas, 135-139, 4050-600 Porto, Portugal; Department of Clinical Epidemiology, Predictive Medicine and Public Health of the University of Porto Medical School, Alameda Prof. Hernâni Monteiro, 4200-319 Porto, Portugal; Ponte de Lima Hospital, Largo Conde de Bertiandos, 4990-041 Ponte de Lima, Portugal; Beatriz Ângelo Hospital, Avenida Carlos Teixeira, 3, 2674-514 Loures, Portugal

**Keywords:** Osteoarthritis, Quality of life, Diagnosis

## Abstract

**Background:**

Osteoarthritis is the most common chronic joint disease. In the absence of an effective medical treatment and due to the chronic nature of this condition, an osteoarthritis medical diagnosis may finally result in decreased health-related quality of life. Therefore, the aim of this study was to measure the impact of the osteoarthritis medical labelling on physical and mental health-related quality of life.

**Methods:**

Subjects (n = 1132, 58.7% women) were approached as participants of an urban population-based cohort (EPIPorto). Self-reported information on previous diagnosis of knee, hip or hand osteoarthritis was obtained and rheumatologists established knee, hip or hand osteoarthritis clinical diagnosis in symptomatic individuals. Physical and mental dimensions of health-related quality of life were evaluated using the self-administered Medical Outcomes Study: 36-Item Short Form Survey. Crude and adjusted linear regression coefficients (beta) and the corresponding 95% confidence intervals (95% CI) were computed to estimate the associations between being labelled as an osteoarthritis case and health-related quality of life.

**Results:**

Regardless of disease medical labelling, individuals with osteoarthritis scored significantly lower physical health-related quality of life when compared to those without joint disease (knee_unexposed_: beta = −5.3, 95% CI: −7.6, −3.1; knee_exposed_: beta = −6.0, 95% CI: −8.4, −3.7; hip_unexposed_: beta = −6.0, 95% CI: −9.8, −2.3; hip_exposed_: beta = −11.0, 95% CI: −15.6, −6.4; hand_unexposed_: beta = −4.3, 95% CI: −6.5, −2.0; hand_exposed_: beta = −4.3, 95% CI: −6.6, −2.1). The same was not observed regarding mental health-related quality of life. Among subjects with clinically confirmed osteoarthritis, the medical labelling of this joint disease was not significantly associated to health-related quality of life.

**Conclusions:**

The labelling of knee, hip and hand osteoarthritis diagnosis may not add specific benefit to osteoarthritis patients in terms of its capability to improve health-related quality of life.

## Background

Musculoskeletal conditions are a major contributor to the burden of disease worldwide [1]. These disorders were responsible for 6.8% of the global disability-adjusted life years worldwide [2], with osteoarthritis representing the single most important condition [3]. Due to its high incidence and low case-fatality, osteoarthritis affects a substantial part of the population [4]. Even though the impact of osteoarthritis on individuals (impairment of daily activities and professional life) and society is undeniable [5-9], doubt persists about the usefulness of early diagnosis in modifying prognosis and patient general well-being, since most non-invasive management approaches show low efficacy or poor adherence [10,11].

Current guidelines emphasize health-related quality of life as a priority in healthcare services, particularly as part of chronic disease management [12]. Quality of life is a personal and autonomous concept that is influenced by social and emotional contexts and not simply explained by the absence of physical symptoms or disease [13,14]. Accordingly, it is plausible that the impact of knowing the name and the expected consequences of a disease may contribute to modify the well-being and welfare judgments and, consequently, the overall perceived health-related quality of life.

Previous research on health-related quality of life suggested that diagnostic labelling affects the self-perception of physical and mental health status regardless of objective pathophysiological changes or previous knowledge about the presence of a certain condition without symptom history [15-18]. The medical labelling of a long-standing chronic disease with suboptimal management effectiveness, as it is expected for osteoarthritis, might impair health-related quality of life without providing the additional benefit to offset it [19]. However, up to the present, the effect of osteoarthritis medical labelling on health-related quality of life remains unknown.

Therefore, using data from a community-based sample of adults, we compared the physical and mental health-related quality of life between previously diagnosed (labelled) and undiagnosed (not labelled) osteoarthritis patients, also using subjects without joint disease as reference.

## Methods

### Participants

The EPIPorto study is a population-based cohort of adult inhabitants of the city of Porto (Portugal). Recruitment and baseline data collection occurred between 1999 and 2003 and have already been described elsewhere [20]. Briefly, subjects were selected by random digit dialling, considering households as the sampling frame. When a household was identified all residents were described by sex and age. One of those residents (aged 18 or older) was randomly selected as the respondent. If the invited resident refused, there was no replacement. A visit to the Medical School was scheduled by telephone, according to the convenience of the participants. Baseline participation proportion was 70% (n = 2485). This investigation was based on cross-sectional data collected at an evaluation of the EPIPorto study carried out between 2005 and 2008 and included 1132 (58.7% women) adults with complete information for all variables of interest. Individuals involved in this study were more frequently women, were on average younger, were more educated, had lower body mass index and scored higher in the physical dimension of health-related quality of life when compared with the remaining cohort participants at baseline.

### Data collection

#### Knee, hip and hand osteoarthritis medical labelling (exposure)

Subjects were classified according to the following sources of information: a) self-reported osteoarthritis; and b) clinical osteoarthritis diagnosis at the moment of evaluation.

- *Self-reported osteoarthritis (labelling)*

Before asking individuals about joint pain or scheduling clinical assessment, participants were asked if they had ever had a medical diagnosis of osteoarthritis (“has a doctor ever diagnosed you with knee/hip/hand arthritis?”).

b) *Clinical diagnosis of osteoarthritis at the moment of evaluation*

All subjects were asked if they had ever felt pain in the knee(s), hip(s) or hand(s) not resulting from direct trauma. Those who answered affirmatively were asked a further set of questions about pain and were classified as having clinically relevant joint pain if they filled one or more of the following criteria: a) at least one medical appointment with exams or medication prescribed in the previous year due to that pain; b) three or more pain episodes in the previous year with a perceived mean intensity equal to or over 60 millimetres (severe pain) in a visual analogue scale; c) one or more pain episodes lasting over one week in the previous six months; or d) one or more pain episodes in the previous month with a perceived mean intensity equal to or over 60 millimetres (severe pain) in a visual analogue scale. This algorithm was previously validated to our sample and has shown to be accurate for screening osteoarthritis of the knee, hip and hand [21].

Those who filled out at least one of those criteria were selected for evaluation by a rheumatologist. After the clinical assessment, participants were classified as having or not having knee, hip or hand osteoarthritis according to the American College of Rheumatology Clinical Classification Criteria for Osteoarthritis of the knee [22], hip [23] and hand [24].

Participants were then classified into one of the following three groups:

1. *Subjects with clinical osteoarthritis but without previous disease label*

Subjects who were classified as having clinical osteoarthritis in the rheumatologic assessment and who were previously unaware of the disease were considered “unexposed” to the diagnosis labelling.

2) *Subjects with clinical osteoarthritis and with a previous disease label*

Participants who were classified in the clinical assessment as having osteoarthritis and who had previously reported that they had a diagnosis of osteoarthritis were considered “exposed” due to their awareness (medical labelling).

3) *Subjects without evidence of significant joint pain or disease*

Since the previous groups consist of individuals with clinically relevant disease, we identified the need to obtain a baseline estimate for health-related quality of life in the target population. For that we used the group composed by all subjects who did not fill at least one of the above-mentioned criteria for significant joint pain or who reported significant pain but were considered free from clinically significant disease by the rheumatologist.

#### Physical and mental dimensions of health-related quality of life (outcomes)

The physical and mental dimensions of health-related quality of life were assessed using the self-administered Medical Outcomes Study 36-Item Short Form Survey (SF-36), which was previously validated for the Portuguese population [25]. All participants answered the SF-36 prior to awareness of their eligibility for the rheumatologist appointment.

The construction of the physical and mental health-related quality of life dimensions was divided in three main steps. In the first step, the eight sub-dimensions of the self-administered SF-36 Portuguese version were normalized (z-scores: mean = 0; standard-deviation = 1) using the means and the standard-deviations obtained in the population of Porto in each sub-dimension of SF-36. In the second step, the eight sub-dimensions were aggregated using the coefficients obtained by principal components analysis. Finally, in the third step, the two SF-36 main components (physical and mental health-related quality of life) were normalized considering mean = 50 and standard-deviation = 10. In both physical and mental dimensions of health-related quality of life, higher values correspond to better levels of functioning and well-being [4,25].

#### Potential confounders

Individual and clinical characteristics that might be causally related both to osteoarthritis labelling and to health-related quality of life were collected.

Demographic and socioeconomic information was collected by face-to-face interviews and included data on sex, age (65 or less; more than 65 years of age) and education (12 or less; more than 12 completed schooling years).

Participants were asked whether in the previous month they had felt knee, hip or hand pain (“during the past month did you experience knee/hip/hand pain?”). Those reporting knee, hip or hand pain in the preceding month were asked about the intensity of knee, hip and hand pain using a visual analogue scale (range: 0–100 millimetres). Subjects were then grouped according to the severity of pain as follows: 40 or less (no pain to mild pain); more than 40 millimetres (moderate to severe pain).

Data on a wide spectrum of self-reported comorbidities (cardiovascular, respiratory, endocrine, cancer, and rheumatic diseases other than osteoarthritis) were collected. Subjects were grouped as not having any comorbid condition or as having at least one comorbid condition.

Weight and height were obtained while the individuals stood barefoot in light indoor clothing. Weight was measured to the nearest tenth of kilogram (Tanita® bioimpedance scales) and height was measured in centimetres, to the nearest tenth with a portable stadiometer (Seca®). Body mass index was calculated dividing the weight (kilograms) by the squared height (metres) and participants were grouped as follows: less than 30 (not obese); 30 or more kg/m^2^ (obese).

### Data analysis

For each anatomical site (knee, hip or hand), individuals were grouped according to the following criteria: a) subjects with clinical osteoarthritis but without previous disease label; b) subjects with clinical osteoarthritis and with a previous disease label; and c) subjects without evidence of significant joint pain or disease.

Participants’ characteristics are presented according to clinically-ascertained and self-reported osteoarthritis in each anatomical site (knee, hip and hand). All categorical variables are presented as counts and proportions and normally distributed continuous variables are presented as means and standard-deviations. Proportions were compared using chi-square tests and continuous variables were compared using Student’s t-test or the One-way Analysis of Variance (ANOVA).

Crude and adjusted (for sex, age, education, body mass index, intensity of pain and clinical comorbidities) linear regression coefficients (β) and the corresponding 95% confidence intervals (95% CI) were computed to estimate the associations between osteoarthritis medical labelling (in the knee, hip and hand) and physical and mental health-related quality of life.

Statistical analyses were performed using Stata® version 11.2 for Windows (Stata Corp. LP, College Station, Texas, USA).

### Ethics

The EPIPorto study was approved by the Ethics Committee of Hospital of São João and the University of Porto Medical School (Porto). Written informed consent in accordance with the 1964 Declaration of Helsinki (and its later amendments) was obtained from all participants.

## Results

### Osteoarthritis labelling (knee, hip or hand)

Table 1 summarizes the characteristics of participants according to clinical evidence and labelling of osteoarthritis in the knee, hip or hand. Among individuals with clinically confirmed osteoarthritis, patients with previous disease labelling were more frequently women, had higher body mass index and reported more severe stages of joint-related pain when compared to those without previous disease labelling.

**Table 1:** **Participants’ characteristics by anatomical site (knee, hip and hand) and according to clinical evidence and labelling of osteoarthritis**

**Anatomical site**	**Total**	**Absence of joint disease**	**With OA but not labelled with OA**	**With OA and labelled with OA**		
	**n (%)**	**n (%)**	**n (%)**	**n (%)**	**p** ^*^	**p** ^**†**^
**Knee (n = 1040)**		906 (87.1)	58 (5.6)	76 (7.3)		
**Sex**						
Female	588 (56.5)	486 (53.6)	42 (72.4)	60 (79.0)	p < 0.001	0.379
Male	452 (43.5)	420 (46.4)	16 (27.6)	16 (21.1)		
**Age**						
≤65 years	778 (74.8)	722 (79.7)	28 (48.3)	28 (36.8)	p < 0.001	0.145
>65 years	262 (25.2)	184 (20.3)	30 (51.7)	48 (63.2)		
**Education**						
≤12 years	693 (66.6)	572 (63.1)	51 (87.9)	70 (92.1)	p < 0.001	0.419
>12 years	347 (33.4)	334 (36.9)	7 (12.1)	6 (7.9)		
**BMI**						
<30 kg/m^2^	830 (79.8)	749 (82.7)	42 (72.4)	39 (51.3)	p < 0.001	0.013
≥30 kg/m^2^	210 (20.2)	157 (17.3)	16 (27.6)	37 (48.7)		
**Intensity of pain**						
≤40 mm	922 (88.6)	868 (95.8)	33 (56.9)	21 (27.6)	p < 0.001	0.001
>40 mm	118 (11.4)	38 (4.2)	25 (43.1)	55 (72.4)		
**Comorbidities**						
No comorbidities	330 (31.7)	314 (34.7)	8 (13.8)	8 (10.5)	p < 0.001	0.563
At least one comorbidity	710 (68.3)	592 (65.3)	50 (86.2)	68 (89.5)		
**Hip (n = 941)**		906 (96.3)	20 (2.1)	15 (1.6)		
**Sex**						
Female	510 (54.2)	486 (53.6)	11 (55.0)	13 (86.7)	0.039	0.046
Male	431 (45.8)	420 (46.4)	9 (45.0)	2 (13.3)		
**Age**						
≤65 years	735 (78.1)	722 (79.7)	9 (45.0)	11 (73.3)	p < 0.001	0.094
>65 years	206 (21.9)	184 (20.3)	11 (55.0)	4 (26.7)		
**Education**						
≤12 years	600 (66.4)	572 (63.1)	16 (80.0)	12 (80.0)	0.126	0.999
>12 years	341 (36.2)	334 (36.9)	4 (20.0)	3 (20.0)		
**BMI**						
<30 kg/m^2^	768 (81.6)	749 (82.7)	16 (80.0)	3 (20.0)	p < 0.001	p < 0.001
≥30 kg/m^2^	173 (18.4)	157 (17.3)	4 (20.0)	12 (80.0)		
**Intensity of pain**						
≤40 mm	897 (95.3)	883 (97.5)	10 (50.0)	4 (26.7)	p < 0.001	0.163
>40 mm	44 (4.7)	23 (2.5)	10 (50.0)	11 (73.3)		
**Comorbidities**						
No comorbidities	321 (34.1)	314 (34.7)	6 (30.0)	1 (6.7)	0.071	0.088
At least one comorbidity	620 (65.9)	592 (65.3)	14 (70.0)	14 (93.3)		
**Hand (n = 1040)**		906 (87.1)	56 (5.4)	78 (7.5)		
**Sex**						
Female	601 (57.8)	486 (53.6)	43 (76.8)	72 (92.3)	p < 0.001	0.011
Male	439 (42.7)	420 (46.4)	13 (23.2)	6 (7.7)		
**Age**						
≤65 years	780 (75.0)	722 (79.7)	23 (41.2)	35 (44.9)	p < 0.001	0.502
>65 years	260 (25.0)	184 (20.3)	33 (58.9)	43 (55.1)		
**Education**						
≤12 years	691 (66.4)	572 (63.1)	47 (83.9)	72 (92.3)	p < 0.001	0.129
>12 years	349 (33.6)	334 (36.9)	9 (16.1)	6 (7.7)		
**BMI**						
<30 kg/m^2^	836 (80.4)	749 (82.7)	39 (69.6)	48 (61.5)	p < 0.001	0.332
≥30 kg/m^2^	204 (19.6)	157 (17.3)	17 (30.4)	30 (38.5)		
**Intensity of pain**						
≤40 mm	945 (90.9)	874 (96.5)	40 (71.4)	31 (39.7)	p < 0.001	p < 0.001
>40 mm	95 (9.1)	32 (3.5)	16 (28.6)	47 (60.3)		
**Clinical comorbidities**						
No comorbidities	334 (32.1)	314 (34.7)	9 (16.1)	11 (14.1)	p < 0.001	0.752
At least one comorbidity	706 (67.9)	592 (65.3)	47 (83.9)	67 (85.9)		

Legend: *OA*: Osteoarthritis; *BMI*: Body mass index; *kg*: Kilogram; *m*: Metres; *mm*: Millimetres.

^*^Comparison between the following groups: absence of joint disease vs. with OA but not labelled with OA vs. with OA and labelled with OA.

^**†**^Comparison between the following groups: with OA but not labelled with OA vs. with OA and labelled with OA.

### Physical and mental dimensions of health-related quality of life

Table 2 presents crude and adjusted linear regression coefficients to quantify the associations between the osteoarthritis medical labelling (knee, hip and hand) and the physical and mental dimensions of health-related quality of life, taking into account two different reference categories: a) individuals with no evidence of significant chronic joint pain; and b) individuals with osteoarthritis but not previously aware of their condition.

**Table 2:** **Linear regression coefficients and the corresponding 95% confidence intervals for the associations between the osteoarthritis medical labelling and the physical and mental dimensions of health-related quality of life in the knee, hip and hand**

**Anatomical site**	**Physical HRQOL**				**Mental HRQOL**			
	**Mean (SD)**	**p**	**Crude β (95% CI)**	**Adjusted β** ^*^ **(95% CI)**	**Mean (SD)**	**p**	**Crude β (95% CI)**	**Adjusted β** ^*^ **(95% CI)**
**Knee**								
Absence of joint disease	52.8 (8.7)	0.148	Ref 1	Ref 1	50.5 (9.8)	0.658	Ref 1	Ref 1
With OA but not labelled with OA	42.3 (10.4)		−10.5 (−12.8, −8.1)	−5.3 (−7.6, −3.1)	47.6 (10.5)		−2.9 (−5.5, −0.3)	−2.1 (−4.8, 0.6)
With OA and labelled with OA	38.8 (8.4)		−14.0 (−16.0, −11.9)	−6.0 (−8.4, −3.7)	46.1 (10.2)		−4.5 (−6.8, −2.1)	−3.5 (−6.3, −0.7)
With OA but not labelled with OA	42.3 (10.4)	0.034	Ref 2	Ref 2	47.6 (10.5)	0.393	Ref 2	Ref 2
With OA and labelled with OA	38.8 (8.4)		3.5 (0.3, 6.7)	1.2 (−1.9, 4.3)	46.1 (10.2)		1.5 (−2.0, 5.1)	1.5 (−2.2, 5.2)
**Hip**								
Absence of joint disease	52.8 (8.7)	0.538	Ref 1	Ref 1	50.5 (9.8)	0.125	Ref 1	Ref 1
With OA but not labelled with OA	42.3 (9.1)		−10.6 (−14.4, −6.7)	−6.0 (−9.8, −2.3)	49.0 (12.3)		−1.5 (−5.9, 2.9)	−1.2 (−5.8, 3.3)
With OA and labelled with OA	32.4 (7.0)		−20.4 (−24.9, −16.0)	−11.0 (−15.6, −6.4)	44.1 (12.6)		−6.4 (−11.5, −1.4)	−5.0 (−10.6, 0.6)
With OA but not labelled with OA	42.3 (9.1)	0.001	Ref 2	Ref 2	49.0 (12.3)	0.253	Ref 2	Ref 2
With OA and labelled with OA	32.4 (7.0)		9.9 (4.1, 15.6)	5.5 (−1.1, 12.1)	44.1 (12.6)		4.9 (−3.7, 13.6)	4.7 (−6.9, 16.2)
**Hand**								
Absence of joint disease	52.8 (8.7)	0.325	Ref 1	Ref 1	50.5 (9.8)	0.169	Ref 1	Ref 1
With OA but not labelled with OA	43.7 (9.3)		−9.1 (−11.5, −6.7)	−4.3 (−6.5, −2.0)	49.3 (10.7)		−1.3 (−3.9, 1.4)	−0.5 (−3.3, 2.3)
With OA and labelled with OA	41.4 (9.8)		−11.4 (−13.5, −9.4)	−4.3 (−6.6, −2.1)	46.4 (11.2)		−4.1 (−6.4, −1.8)	−2.3 (−5.0, 0.4)
With OA but not labelled with OA	43.7 (9.3)	0.172	Ref 2	Ref 2	49.3 (10.7)	0.144	Ref 2	Ref 2
With OA and labelled with OA	41.4 (9.8)		2.3 (−1.0, 5.6)	0.0 (−3.1, 3.1)	46.4 (11.2)		2.8 (−1.0, 6.6)	1.2 (−2.8, 5.2)

Legend: *HRQOL*: Health-related quality of life; *SD*: Standard-deviation; β: linear regression coefficient; 95% *CI*: 95% Confidence interval; *OA*: Osteoarthritis.

^*^Adjusted for sex (female; male), age (<65; ≥65 years), education (≤12; >12 years), body mass index (<30; ≥30 kg/m^2^), intensity of pain (≤40; >40 millimetres) and clinical comorbidities (no comorbidities; at least one comorbidity).

Ref 1: this reference group comprised individuals who were not diagnosed with osteoarthritis in any anatomical region in study.

Ref 2: this reference group comprised individuals who were diagnosed with osteoarthritis but were not previously labelled with this joint disease.

#### Physical dimension of health-related quality of life

Regardless of disease labelling, subjects who were clinically diagnosed with knee, hip or hand osteoarthritis perceived lower physical health-related quality of life when compared to those without osteoarthritis. These associations remained statistically significant after adjustment for sex, age, education, body mass index, intensity of pain and clinical comorbidities (knee_unexposed_: β = −5.3, 95% CI: −7.6, −3.1; knee_exposed_: β = −6.0, 95% CI: −8.4, −3.7; hip_unexposed_: β = −6.0, 95% CI: −9.8, −2.3; hip_exposed_: β = −11.0, 95% CI: −15.6, −6.4; hand_unexposed_: β = −4.3, 95% CI: −6.5, −2.0; hand_exposed_: β = −4.3, 95% CI: −6.6, −2.1).

Among individuals with clinically confirmed osteoarthritis, no significant associations were found between the awareness of having knee, hip or hand osteoarthritis and the physical dimension of health-related quality of life, even after adjustment for confounders.

#### Mental dimension of health-related quality of life

After adjustment for sex, age, education, body mass index, intensity of pain and clinical comorbidities, we observed that osteoarthritis presence (medically labelled or not) did not change significantly mental health-related quality of life when the comparison group was composed by individuals without joint disease (knee_unexposed_: β = −2.1, 95% CI: −4.8, 0.6; knee_exposed_: β = −3.5, 95% CI: −6.3, −0.7; hip_unexposed_: β = −1.2, 95% CI: −5.8, 3.3; hip_exposed_: β = −5.0, 95% CI: −10.6, 0.6; hand_unexposed_: β = −0.5, 95% CI: −3.3, 2.3; hand_exposed_: β = −2.3, 95% CI: −5.0, 0.4).

Among individuals with clinically confirmed joint disease, we did not observe any significant association between the labelling of knee, hip or hand osteoarthritis and the mental dimension of health-related quality of life, even after adjustment for confounders.

## Discussion

Our findings suggest that, compared to individuals with no evidence of significant chronic joint pain, osteoarthritis significantly reduces physical health-related quality of life regardless of its medical labelling. However, among individuals with clinically confirmed disease, the labelling of knee, hip or hand osteoarthritis did not play a major role in physical or mental health-related quality of life scores.

Previous research has shown that osteoarthritis patients frequently score lower in health-related quality of life when compared to those without this condition [26-28]. Beyond the negative emotional impact of living with a chronic joint disease, osteoarthritis affects joints that are crucial to the performance of daily activities [29,30], which was underlined by our findings showing that a worse physical health-related quality of life occurs when osteoarthritis is present (compared to those with no evidence of osteoarthritis), independently of the joint considered.

Regarding the physical dimension of well-being, evidence has been consistent in showing that disease labelling negatively influences quality of life [17,18,31]. Cancer labelling has been particularly investigated, showing that the awareness of disease presence and severity significantly worsened the perception of health-related quality of life [32-35]. Nevertheless, among individuals with clinical confirmed osteoarthritis, disease labelling did not significantly interfere with health-related quality of life. Diagnosis establishment is expected to positively change the natural history of the condition, since it is expected to guide clinical practice into a better treatment and management of the disease. Osteoarthritis is a particular case because the available conservative treatments have insufficient effectiveness, and its surgical alternative (joint replacement surgery) is usually restricted to more severe cases of disease and has low acceptability in some settings [36]. Comparatively to other chronic diseases, osteoarthritis labelling may have lower potential to modify physical health-related quality of life particularly because without an obvious compensatory treatment benefit, the role of diagnosis may exclusively contribute to the amplification of the vigilance and concern about the joint disease-related symptoms [37]. The fact that osteoarthritis has virtually null case-fatality when compared to other conditions such as cancer may also account for the negligible effect we have found in this study.

In relation to mental health-related quality of life, we did not observe a consistent effect of osteoarthritis labelling on this dimension of well-being, whether the reference group was composed by subjects without joint disease or by osteoarthritis patients not previously aware of the diagnosis. On the one hand, osteoarthritis labelling might trigger negative cognitions and emotions due to the awareness of living with a chronic disease whose effectiveness of treatments is doubtful [38,39]. On the other hand, osteoarthritis labelling might have a positive interpretation, i.e., better chronicity than fatality. The balance between these two opposite meanings of osteoarthritis labelling is a probable explanation to the absence of significant results regarding mental-health related quality of life, even when the comparison group was composed by subjects without clinically confirmed joint disease.

Active case-finding of osteoarthritis, unlike clinical examination of unspecific complaints, implies the systematic search for one disease by taking advantage of the contacts of previously unaware individuals with the health care services. In clinical practice there is little advantage in searching for the presence of osteoarthritis in the absence of joint pain [40], which is reasonable given that doubt persists about the usefulness of early diagnosis and treatment in modifying prognosis [11,19]. If an effective medical intervention and management of the disease occurred after osteoarthritis diagnosis, the presence of a previous medical labelling should be associated to a significant improvement of health-related quality of life, especially in its physical dimension. However, as suggested by our findings, the labelling of osteoarthritis seems not to provide any specific additional benefit regarding health-related quality of life of patients.

To the best of our knowledge, this is the first study looking at the effect of osteoarthritis medical labelling on physical and mental health-related quality of life, using as reference a group of subjects from the general population without evidence of joint disease. This is a major strength since baseline health-related quality of life may depend on specific characteristics of the source population, particularly in middle-aged individuals. Another major strength of this study is that it provides an ample spectrum of the disease severity because it was based on data collected in a community-based sample. All participants were assessed using the same protocol and were evaluated by a single observer (rheumatologist) using the gold standard method for case ascertainment, which is also a relevant advantage of this research.

Nevertheless, some methodological issues need to be addressed. Recall bias may reduce the validity of the evocation of the information that was used to define preceding label of osteoarthritis. The number of years elapsed between knee, hip or hand osteoarthritis medical labelling and the moment of evaluation of the EPIPorto cohort was analysed as a potential marker of recall bias but its inclusion did not meaningfully change the magnitude of the estimates and was not considered in the multiple linear regression models as covariate. In addition, the validity of self-reported information on previous medical diagnoses was not optimal. Accurate reporting of osteoarthritis medical labelling is dependent on the characteristics of patients as well as on the medical workup used (clinical evaluation and/or radiography). Previous studies have shown that self-reported diagnosis of this type of condition is not commonly congruent with the medical one [41]. In our study, we found that agreement between self-reported and clinical and radiographic osteoarthritis diagnosis in our sample was poor (data not shown). However, independently of the accuracy of self-reported osteoarthritis (our main exposure), our priority was to assess the beliefs of individuals regarding a potential presence of osteoarthritis, whether it was clinically valid or not.

The identification of subjects with osteoarthritis was based on a structured questionnaire where individuals were asked about joint pain: individuals that did not report joint pain were classified as not having osteoarthritis. We assumed that questions about joint pain presence had a negative predictive value of 100%. Although most osteoarthritis patients have joint pain [42], we recognize the possibility of occurrence of false negatives. Additionally, alternative diagnoses of the most common inflammatory joint conditions were inquired (rheumatoid arthritis, ankylosing spondylitis, psoriatic arthritis and systemic lupus erythematosus). As expected, given their epidemiological pattern, the frequency of those diseases was much lower than that of osteoarthritis (below 2%). Thus, we do not expect that these contribute substantially to osteoarthritis misclassification in this community-based sample.

Data on potential confounders of the associations between osteoarthritis medical labelling and health-related quality of life were measured and included as covariates in the multiple linear regression models (sex and age, body mass index, clinical comorbidities – wide spectrum of chronic diseases – and intensity of pain). Since these variables returned significant changes in the magnitude of the estimates and were statistically significant, they remained in the models as confounders. Intensity of pain episodes in the preceding month was used as proxy of osteoarthritis severity. We understand that using diaries of pain in which participants should report the intensity of pain *in loco* was probably the most adequate approach to measure the intensity of retrospective episodes of pain and consequent severity of the disease. However, since previous literature has shown that subjects are able to accurately recall and rate severity of pain or discomfort and that retrospective reports of intensity of pain are sufficiently reliable [43], we believe that our methodological option did not substantially change our main findings.

In addition, both osteoarthritis labelling and health-related quality of life may be influenced by exposure to joint pain treatments [11]. Nevertheless, we did not find any significant difference between exposed and not exposed individuals to osteoarthritis treatments (painkillers and anti-inflammatory drugs, physiotherapy or thermal spa) regarding the physical and mental health-related quality of life scores, independently of the joint in study.

Osteoarthritis presence was clinically evaluated in three different joints: knee, hip and hand. Concomitant osteoarthritis in the remaining sites may confound the associations between labelling in a specific site and quality of life: a subject may be included in the analysis as exposed to labelling regarding one joint and not exposed for the remaining joints. A sensitivity analysis was conducted, by stratifying the estimates according to the number of joints affected. Since changes in the magnitude or direction of associations were not observed, we believe that the number of joints diagnosed with osteoarthritis was not a major drawback to the interpretation of our results.

Finally, the small sample sizes used in the linear regression models may be a methodological limitation, especially when the models were computed using data only from individuals with clinically confirmed osteoarthritis. Sensitivity analyses were performed in order to test the impact in our estimates of the small sample size using the bootstrap method (replications = 100) [44]. We observed a remarkable similarity between the 95% confidence intervals obtained in the original analyses and those computed by bootstrapping, which suggests that the small numbers of participants that were used in the linear regression models did not significantly impair our findings.

## Conclusions

The labelling of knee, hip and hand osteoarthritis diagnosis may not add specific benefit to osteoarthritis patients in terms of its capability to improve health-related quality of life. Our findings may question the usefulness of active case-finding of osteoarthritis during regular contacts of asymptomatic individuals with the health care services.
